# A Critique of Coronavirus

**DOI:** 10.3201/eid2607.201426

**Published:** 2020-07

**Authors:** Elana R. Osen

**Affiliations:** St George’s University Hospitals National Health Service Foundation Trust, London, UK

**Keywords:** viruses, zoonoses, coronavirus, pandemics, infectious diseases, COVID-19, respiratory infections, coronavirus, pandemics, 2019 novel coronavirus disease, coronavirus disease, severe acute respiratory syndrome coronavirus 2, SARS-CoV-2

Why did the quiet descend?

Does this plague not know

that apocalypses come with fanfare,

wails of lamentation,

howls of wayward dogs,

explosive blasts?

Or, maybe, silence.

Just shop-window glass crunching underfoot

puncturing the eerie nothing.

Not quiet.

Never quiet.

Why does the sun still shine?

Can it not see what transpires

from its lofty throne

above the Earth?

Read the room, sun.

Now’s the time for greyscale filter.

Or, maybe, an eclipse.

One last blinding ray of blazing flare

to scorch the land,

to boil the sea,

to serve up *des hommes brûlés*

to whichever vengeful deity

dines with us tonight*.*

Not sunshine.

Never sunshine.

Why can I smell the tulips?

I thought the virus

wiped olfaction from our

paltry list of powers?

Or, maybe, smoke.

You know, from voracious flames

feasting on our foliage and flesh,

the smog of industry,

of mushroom clouds.

Why does that not sting my nostrils?

Not flowers.

Never flowers.

Why does life go on inexorably?

Is Ragnarök not supposed to happen

around now?

Where are the horsemen?

Where are the double gates of Paradise?

What a lame apocalypse:

we’ve been sold a lemon.

Or, maybe, pop culture eschatology

isn’t all it is cracked up to be.

I thought the zombies would be roaming

all my haunts

by now.

Not life.

Never life.

